# Possible synergic action of non-steroidal anti-inflammatory drugs and glucosamine sulfate for the treatment of knee osteoarthritis: a scoping review

**DOI:** 10.1186/s12891-022-06046-6

**Published:** 2022-12-12

**Authors:** Nicola Veronese, Fiona Ecarnot, Sara Cheleschi, Antonella Fioravanti, Stefania Maggi

**Affiliations:** 1 Department of Internal Medicine, Geriatrics Section, University of Palermo, Palermo, Italy; 2 Research Unit EA3920, University of Franche-Comté, 25000 Besançon, France; 3 Department of Cardiology, University Hospital Besancon, 3 Boulevard Fleming, 25000 Besancon, France; 4 Rheumatology Unit, Department of Medicine, Surgery and Neuroscience, Azienda Ospedaliera Universitaria Senese, Policlinico Le Scotte, 53100 Siena, Italy; 5 National Research Council, Neuroscience Institute, Aging Branch, Padua, Italy

**Keywords:** Celecoxib, Chondroprotective effect, Cyclooxygenase 2 inhibitors, Glucosamine sulfate, Non-steroidal anti-inflammatory drugs, Osteoarthritis, Synergistic effects

## Abstract

**Background:**

Several studies have reported that glucosamine sulfate (GS) can improve knee osteoarthritis (OA) symptomatology. In parallel, the disease-modifying effects of non-steroidal anti-inflammatory drugs (NSAIDs) in knee OA have also been investigated. However, limited literature has reported the combined effect of GS and NSAIDs. The aim of this scoping review is to describe the scope and volume of the literature investigating the potential benefits and synergistic effect of a combination of GS and NSAIDs in patients with knee OA.

**Methods:**

PubMed and Embase were searched for studies published from inception through April 2022, evaluating the effects of the combination of GS and NSAIDs in OA patients, versus either treatment alone. Data are reported narratively.

**Results:**

Five studies were included in this review; 4 were randomized control trials and one was a prospective observational study. The duration of combination treatment was 6 to 12 weeks. The combination was compared to celecoxib in 2 studies, meloxicam in 1, etoricoxib in 1, and a conventional NSAID in 1 (ibuprofen or piroxicam). All 5 studies reported that in patients with knee OA, the combination of GS plus NSAID yielded a significantly greater benefit than single-agent therapy, in terms of outcomes including pain reduction, function, joint stiffness, and markers of inflammatory activity and cartilage degradation.

**Conclusion:**

The 5 studies included in this scoping review all report a significantly greater clinical benefit with a combination of GS plus NSAID compared to either treatment alone. The evidence supports efficacy in reducing pain, improving function, and possibly regulating joint damage. However, further randomized trials with larger sample sizes are warranted to confirm these findings.

**Supplementary Information:**

The online version contains supplementary material available at 10.1186/s12891-022-06046-6.

## Background

Osteoarthritis (OA) is a common degenerative musculoskeletal disorder that causes significant health-related and social problems. The major symptoms of OA include chronic pain, functional impairment, instability, and deformity, which can lead to impaired quality of life [1, 2]. Recently, data from the 2019 Global Burden of Disease study have shown that the prevalence of OA increased dramatically, from 247.51 million in 1990 to 527.81 million in 2019, at a rate of 113.25%. Over the same period, the global trend in years lived with disability due to OA increased by 114.5% [3].

Current treatment options for OA include non-pharmacological, pharmacological, and surgical interventions, based on disease severity and joint site [4]. Among the various pharmacological interventions, the European Society for Clinical and Economic Aspects of Osteoporosis, Osteoarthritis and Musculoskeletal Diseases (ESCEO) algorithm recommends the use of symptomatic slow-acting drugs for osteoarthritis (SYSADOAs). This includes prescription-grade glucosamine sulfate (pGS) or chondroitin sulfate (CS) as first-line therapy for long-term background treatment, and paracetamol as short-term rescue medication [5]. Although other guidelines, for example, those from AAOS [6], ACR [7], NICE [8], OARSI [9] do not recommend the use of GS or CS as first-line therapy for OA, we believe that available data on efficacy and safety of pGS support its use as a first-line background therapy, particularly as an alternative to widely used drugs, such as paracetamol, which has been reported to have limited clinical efficacy [10] with some safety concerns [11].

Glucosamine, a natural amino monosaccharide, is a normal constituent of glycosaminoglycans. It is present in the extracellular matrix of the cartilage, in the synovial fluid, and in higher quantities in articular cartilage [12]. Glucosamine sulfate (GS) is both an oral supplement of glucosamine and is registered as a drug when prescription-grade. It is thought to have anti-inflammatory and anti-apoptotic effects on articular cartilage and bone, and reportedly also has prebiotic properties [13–15]. Not all guidelines for the treatment of OA have taken into consideration the distinction between studies that used prescription drugs, and studies that used food supplements, or the distinction between studies carried out with GS and Glucosamine hydrochloride (characterized by a low pharmacokinetic profile compared to GS).

International guidelines [7, 9, 16] provide (strong or conditional) recommendations for the usage of oral non-steroidal anti-inflammatory drugs (NSAIDs) for patients with knee, hip, and/or hand OA. NSAIDs can be categorized into conventional NSAIDs and selective cyclooxygenase 2 inhibitors (COX-2) [16], with existing evidence that certain drugs in the NSAID class may have a more favourable safety profile than others. In this context, it is suggested that selective COX-2 inhibitors be preferred in individuals with gastrointestinal (GI) comorbidities [7, 9, 16] because they have a more favourable upper GI safety profile than non-selective NSAIDs. Oral NSAID therapy is the mainstay of pharmacological management in knee OA (KOA) [7], although few international OA guidelines address the relative merits of different drugs among the selective and non-selective NSAIDs. There is nonetheless consensus that NSAID dosage should be as low as possible, and treatment duration should be as short as possible [7, 9]. The ESCEO clearly recommends the use of oral NSAIDs (selective or non-selective) in the second step of its management algorithm, when subjects still have pain or functional limitation after background therapy with SYSADOAs. In the ESCEO algorithm [5], it is suggested that NSAIDs be used only intermittently for longer cycles, and the appropriate molecule should be selected based on the patient’s risk profile.

Celecoxib (a COX-2 inhibitor) may be considered the preferred oral NSAID, due to its favourable balance between good short-term efficacy in OA and a lower propensity for toxicity, especially at the GI and cardiovascular (CV) levels [5]. In a pre-specified secondary analysis of the PRECISION trial, Obeid et al. reported a significantly lower risk of the composite cardiorenal outcome (adjudicated renal event, hospitalization for congestive heart failure or for hypertension) with celecoxib as compared to ibuprofen, and a trend towards a lower risk as compared with naproxen, highlighting the more favourable cardiorenal safety profile of celecoxib [17]. Another clinical trial reported a significantly lower risk of clinically significant upper or lower GI events with celecoxib, as compared to a combination of diclofenac plus omeprazole [18]. Recent evidence further suggests that COX-2 inhibitors may exhibit disease-modifying effects, in addition to their analgesic and anti-inflammatory properties [19–22]. Several in vitro and in vivo studies have reported that celecoxib in particular demonstrates disease-modifying OA effects [19, 23–25]. A recent in vitro study also suggested that a combination of pGS and celecoxib provides a synergistic chondroprotective and anti-inflammatory effect on chondrocyte cultures [26].

To provide a complete understanding of the possible synergistic effects of the combination of GS and NSAIDs, we conducted a scoping review. A scoping review is an ideal tool to assess the extent of the current literature in terms of volume and scope, with a view to summarizing the available body of research evidence. It applies the same rigorous methods as a systematic review for the selection and analysis of potentially eligible publications in the field of interest [27]. Finally, it provides a descriptive overview of the relevant literature, albeit without evaluating individual studies or synthesizing evidence from different studies [28].

Therefore, the objective of this scoping review was to systematically review the literature and summarize available evidence regarding the possible synergistic effect of a combination of GS plus NSAIDs, in reducing pain and improving function, and potentially regulating cartilage damage, as compared to either GS or NSAIDs alone, in people affected by KOA.

## Methods

### Study design

For this scoping review, a comprehensive literature search was performed to identify all studies related to the question “Does a synergistic action of NSAIDs and GS exist in human beings for the treatment knee osteoarthritis?” The scoping review was designed using the following steps: (1) Convening a research team comprised of health care professionals, and experts in the field of osteoarthritis and research analysis; (2) developing a search strategy; (3) determining the inclusion and exclusion criteria; (4) identifying the relevant studies through a database search; (5) screening and study selection; (6) data extraction and charting; (7) summarizing and reporting the results. This report of the scoping review results follows the Preferred Reporting Items for Systematic reviews and Meta-Analyses (PRISMA) extension for Scoping Reviews. The review followed a pre-planned protocol, which is available from the corresponding author on reasonable written request.

### Search strategy

The literature search was run in MEDLINE, Cochrane Central, and Embase. The following key search terms were combined: ‘Glucosamine sulfate’, ‘Non-steroidal anti-inflammatory drugs’, ‘Celecoxib’, ‘Cyclooxygenase 2 inhibitors’, and ‘Human beings’. Medical subject headings (MeSH) and other relevant keywords (for e.g., NSAIDs, 2-Amino-2-Deoxyglucose, etc.) were used depending on the database. The search terms were combined using Boolean operators such as AND/OR to extract the appropriate results. The search included all studies available in the databases from inception through April 2022 and was restricted to literature published in the English language. The detailed search strategy and search strings are presented in Additional file 1.

### Eligibility criteria

We included studies that met the following inclusion criteria: Randomized controlled trials (RCTs), prospective cohort studies, non-randomized controlled trials, quasi-experimental studies, retrospective cohort studies or case–control studies investigating the concomitant use of GS plus NSAIDs compared to either GS or NSAIDs alone. We excluded studies not published in English, as well as reviews, case reports, consensus statements, and study protocols. Non-human studies and studies reporting a head-to-head comparison of NSAIDs vs GS were also excluded.

### Data extraction

**Fig. 1:**
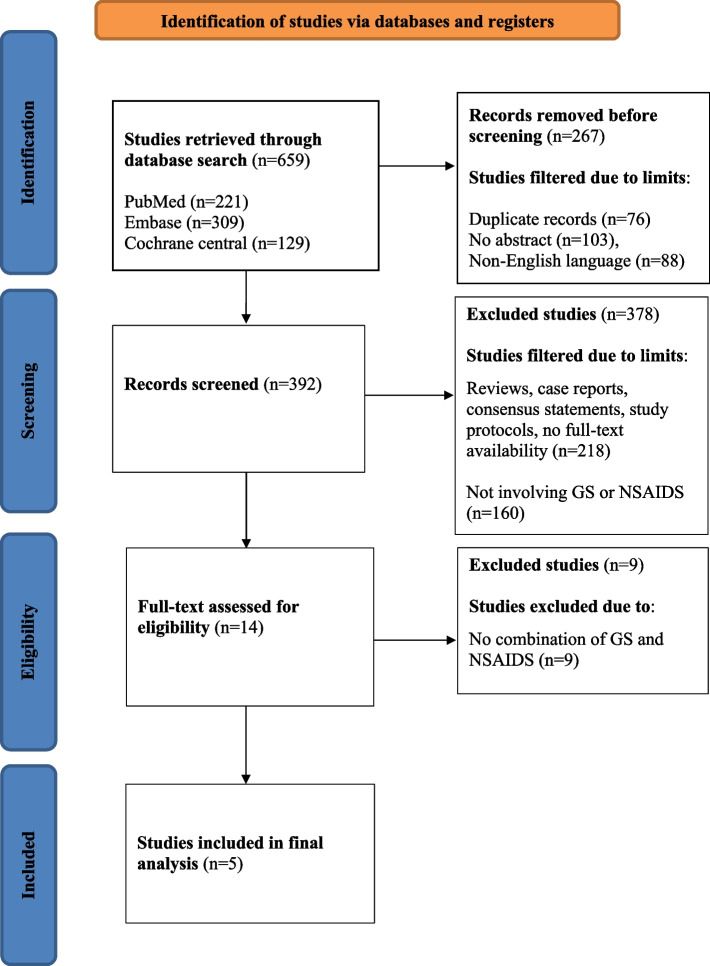
Preferred reporting items for systematic reviews and meta-analyses (PRISMA) flow chart

In the first stage, all the titles and abstracts resulting from the literature search were reviewed by two independent authors (NV and SM). Duplicate studies, studies not published in English, and studies without abstracts were removed. In the next stage, the inclusion and exclusion criteria were verified, and articles were filtered as per the eligibility criteria, by two independent authors (NV and SM). In case of disagreement, a consensus was obtained after a thorough discussion. The full text of the eligible articles were retrieved, and the reference lists of the full-text articles were manually checked to ensure no relevant eligible studies were overlooked. Then, the following key information was extracted and summarized: name of the study, date of publication, first author, population included, study design, sample size, medications taken in the intervention and control groups, and main findings. Further details of the data extraction are presented in Fig. 1.

## Results

### Literature search

The database search yielded a total of 659 articles. Of these, 267 studies were excluded because they were either published in a language other than English or were present in duplicate. The title and abstracts of the remaining 392 articles were screened; 378 were excluded due to the nature of the study design (reviews, case reports, consensus statements, or study protocols) or because they did not involve either GS or NSAIDs. After screening, 14 full-text articles were assessed against the eligibility criteria, of which nine were excluded. Finally, 5 studies (4 RCTs and 1 prospective observational study) were included in the scoping review [29–33] (Fig. 1). A summary of the findings of the 5 included studies is presented in Table 1.

**Table 1:** Descriptive and main findings of the studies included

Study, year, country [Ref]	Population	Design of the study	Sample size	Intervention group/ Treatment group	Control group	Treatment duration	Main findings
Amuzadeh, 2014, Iran [29]	Knee OA (1–2 grade)	Prospective Observational	60	Celecoxib 200 mg/day and GS 500 mg three times/daily	Celecoxib 200 mg/day	8 weeks	Concomitant use of GS with celecoxib is more effective than celecoxib alone in reducing morning stiffness (p = 0.000) and reducing pain & improving function (WOMAC Index 95% CI, Z = -4.891, p = 0.000) in women with early knee OA
Lu, 2019, China [30]	OA	RCT	88	Meloxicam 7.5 mg/day and GS 500 mg three times/daily	Meloxicam 7.6 mg/day	8 weeks	Concomitant use of GS and meloxicam is more effective than meloxicam alone in reducing the clinical symptoms (VAS, 2.35 ± 1.08; LI, 4.78 ± 3.34; p < 0.05) and serum biomarkers (p < 0.05) of bone resorption and bone degradation in patients with OA
Deng, 2015, China [31]	Knee OA	RCT	120	Celecoxib 200 mg/day + GS 500 mg 3 times/day	Celecoxib 200 mg/day	8 weeks	The combination of celecoxib with GS effectively reduces immune inflammatory response, oxidative stress damage, and joint pain (VAS, 2.36 ± 0.52; WOMAC, 40.35 ± 2.36; and LKSS, 87.29 ± 10.38; p < 0.05] associated with KOA
Sun, 2020,China [32]	Knee OA	RCT	80	GS 500 mg three times/daily + etoricoxib 60 mg/day	Etoricoxib 60 mg/day	6 weeks	GS combined with etoricoxib may reduce the expression of JNK and Wnt5a to inhibit the secretion of MMP, and then slow down the degradation of cartilage matrix, leading to an improvement in clinical scores (92.42%, p = 0.000)
Selvan, 2012, India [33]	Knee OA	RCT	100	GS 500 mg three times/daily	GS 500 mg three times/daily + NSAID	12 weeks	GS and NSAIDs group had significant improvement in pain (VAS, 1.12 [0.84–1.41], p < 0.01) (WOMAC, 5.37 [4.97–5.78], p < 0.01), physical function (8.20 [7.51–8.89], p < 0*.*01) and stiffness (2.23 [2.21–2.44], p < 0.01) compared to GS alone

OA, Osteoarthritis; GS, Glucosamine sulfate; WOMAC, Western Ontario and McMaster Universities Arthritis Index score; VAS, Visual Analogue Scale; LI, Lequesne Index; LKSS, Lysholm Knee Score Scale; RCT, Randomized Control Trial; JNK, C-Jun N-terminal kinase; MMP, Matrix Metalloproteinase; Wnt5a, Wnt Family Member 5a

#### Evidence of the synergistic and beneficial effects of the combination of GS and NSAIDs

All 5 studies reported a significant benefit of GS plus NSAIDs compared to GS or NSAID alone in OA. There were no other concomitant interventions in the studies included.

Four RCTs [30–33] were designed to evaluate the effect of a combination of GS plus NSAIDs versus either agent alone in patients with KOA were included in the scoping review.

Lu Zhijun et al. compared GS plus meloxicam vs meloxicam alone in 88 patients in OA; 20 males and 24 females aged 62 ± 3 years in the treatment group, and 21 males and 23 females aged 60 ± 3 years in the control group [30]. The course of disease was around 8 years on average in both groups. They reported a significant improvement in the primary outcome of clinical symptoms, evaluated by the Lequesne index, in the GS + meloxicam group. Pain scores evaluated by the visual analogue scale (VAS) and motor function, assessed by the Lysholm knee joint score, were also significantly improved with combination therapy, as compared to meloxicam alone. Serum biomarkers of disease activity (C-terminal telopeptide type 1 (CTX-I), CTX-II, cartilage oligomeric matrix protein (COMP), and matrix metalloproteinase-3 (MMP-3)) were also significantly lower in the combination therapy group after treatment. In total, overall clinical effectiveness was significantly higher in the intervention group vs the meloxicam alone group (92.7% vs 75%; p = 0.015). Adverse reactions during treatment were mainly gastrointestinal and pruritus, but were of short duration and spontaneously resolved. No adverse reaction required treatment. The authors therefore concluded that treatment with a combination of meloxicam and GS was more effective than meloxicam alone, in reducing the serum markers and clinical symptoms of OA [30].

In the study by Gang Deng et al., 120 patients with KOA were randomly assigned to a combination of GS plus celecoxib versus celecoxib alone for 8 weeks [31]. The population consisted of 72 males and 48 females aged 45 to 75 years (mean 60.5 ± 5.8 years). The disease duration ranged from 6 months to 15 years, and 64 patients had mild disease, 40 had moderate and 19 had severe disease according to the authors. The primary outcome of total effectiveness was significantly higher in the combination therapy group, vs celecoxib alone (93.33% vs 71.66%, p < 0.05). Similarly, the combination of GS and celecoxib yielded statistically significant improvements in inflammatory markers (TNF-α [15.28 ± 3.60 vs 12.56 ± 3.50], IL-1 [47.51 ± 7.32 vs 41.25 ± 7.58], PGE2 [134.64 ± 17.21 vs 121.38 ± 28.68]), oxidative stress parameters such as malondialdehyde (14.54 ± 7.23 vs 8.35 ± 5.10), pain score (VAS [5.34 ± 1.01 vs 2.36 ± 0.52], Western Ontario and McMaster Universities Osteoarthritis Index (WOMAC) score [54.23 ± 3.63 vs 40.35 ± 2.36]), and Lysholm knee joint score [75.63 ± 9.15 vs 87.29 ± 10.38] compared with the control group. During treatment, adverse reactions in the observation group (6 events, 10%) were significantly lower than those in control group (13 events, 21.67%, p < 0.05). These findings suggest a benefit of the combination of GS and celecoxib in inhibiting the progression of OA and improving joint function [31].

The study conducted by Sun et al. compared 40 KOA patients receiving etoricoxib alone (control group, 9 males, 31 females, mean age 62 ± 11 years) to 66 patients receiving a combination of GS plus etoricoxib (experimental group, 14 males, 52 females, mean age 61 ± 10) [32]. Disease course was 3.59 and 3.74 years in the control and experimental groups respectively. In terms of the Kellgren-Lawrence classification, there were 9 cases of grade I, 15 cases of grade II, and 16 cases of grade III. Patients were evaluated for knee function, as assessed using the WOMAC, and for clinical efficacy. Bone metabolism indices, growth factors, inflammatory factors, MMPs, nitric oxide (NO)-induced apoptosis-related factors, and mRNA levels of C-Jun N-terminal kinase (JNK) and Wnt5a were determined. The authors found that compared to monotherapy, combination therapy yielded a significant improvement in total clinical efficacy (92.42% vs. 67.50%, P < 0.0001), and significantly reduced WOMAC pain scores (41.83 ± 4.09 vs 63.57 ± 7.3) and inflammatory markers (IL-1β [51.48 ± 4.89 vs 38.56 ± 3.74], IL-17 [205.38 ± 19.76 vs 276.41 ± 26.11], IL-18 [148.73 ± 13.25 vs 184.67 ± 17.13], TNF-α [52.45 ± 5.02 vs 30.52 ± 2.86], MMP-3 [98.46 ± 9.75 vs 158.37 ± 14.82], MMP-9 [30.26 ± 2.97 vs 45.38 ± 4.62], and MMP-13 [152.43 ± 14.72 vs 193.76 ± 18.69]). Furthermore, combination therapy yielded an improvement in markers of bone metabolism and lowered the expression of JNK and Wnt5a, which inhibited the secretion of MMPs, thereby leading to a decrease in the degradation of the cartilage matrix [32].

The fourth RCT, reported by Selvan et al., evaluated the effectiveness of the combination of GS plus a conventional NSAID (ibuprofen or piroxicam) compared with GS alone in patients with mild to moderate OA recruited through the rheumatology outpatient department [33]. A total of 43 patients were treated with GS alone and 39 GS plus either ibuprofen or piroxicam. The average age of females in the study population was lower than that of male participants overall (47.96 ± 5.09 vs 48.98 ± 8.94 respectively), but the average age of the overall population was not specified. WOMAC and VAS scores were used to evaluate the effectiveness of the combination treatment. There were significantly greater decreases in mean WOMAC pain, stiffness and function scores in the combination therapy group after 12 weeks of treatment (mean difference 5.37 (95% CI: 4.97–5.78, p < 0.01) for pain, 2.23 (95% CI: 2.21–2.44, p < 0.01) for stiffness, and 8.20 (95% CI: 7.51–8.89, p < 0*.*01) for function). There was a significant decrease in mean VAS score in both groups over the treatment period, but the decrease was of significantly greater magnitude in the combination therapy group (p < 0.01). These results confirmed the greater benefit observed with a combination of GS plus a conventional NSAID versus GS alone in mild to moderate OA [33].

Finally, in a prospective observational study by Amuzadeh et al., 30 women ranging in age from 37 to 49 years, with mild KOA treated with celecoxib alone (mean age 45.77 ± 3.42 years) were compared to 30 women treated with a combination of celecoxib and GS (mean age 45.13 ± 3.45 years) [29]. The primary endpoint was the WOMAC index after 8 weeks of treatment. There was a significant reduction in WOMAC pain and morning stiffness scores and a statistically significant improvement in performance (p < 0.0001) in the group receiving combination therapy. The authors concluded that the use of GS and celecoxib in combination is more effective than celecoxib alone in women with early KOA [29].

## Discussion

The aim of this scoping review was to explore the possible synergistic effects of the combination of GS and NSAIDs in the management of OA symptoms. Overall, based on the findings of available publications, it seems that the combination of GS plus NSAIDs could yield greater benefit than either medication alone in terms of clinical outcomes and molecular profiles.

Inadequate pain relief, reduced functional capacity and impaired quality of life are common among patients with KOA [34]. Therefore, the key objective of OA treatment is to provide adequate pain relief and avoid disability. In this regard, oral NSAIDs are widely prescribed for KOA patients and are recommended by recently published guidelines [5, 7, 9].

GS has been reported to have prebiotic properties, in addition to its anti-inflammatory and anti-apoptotic effects on cartilage and bone. It works as a substrate for gut microbiota (i.e. sulfate-reducing bacteria that are implicated in the synthesis of anti-inflammatory compounds). Furthermore, GS is a component of the intestinal mucin that is protected from degradation, thus positively affecting gut permeability and reducing low-grade inflammation [15]. However, despite the use of GS as background therapy, many people with KOA may still suffer from pain and limited function. Therefore, a multimodal treatment approach combining GS with NSAIDS may aid in alleviating symptoms.

In this scoping review, we identified five studies that investigated the potential benefits of co-administration of GS and NSAIDs. In 2 of the 5 studies, celecoxib was used, and in all the studies, the treatment duration was between 6 and 12 weeks. In the study conducted by Sun et al. the prescription-grade crystalline GS (pCGS, Rottapharm Ltd) was used [32], while in the remaining studies, GS was used. Only 2 of the studies included in this review reported data regarding the safety of use of GS; one reported no adverse events requiring treatment, while the other reported fewer adverse events in the observation group than in the control group. In the absence of detailed information about the safety profile of the products used, it is difficult to draw any comparisons or conclusions regarding the potential effects of long-term treatment with GS. This point warrants further investigation in long-term, randomized studies that record adverse events using standardized definitions and validated systems for the classification of severity. All the studies identified in this scoping review reported a significantly greater benefit with a combination of GS plus NSAID, compared to either treatment alone. The results of these studies are corroborated by a recent in vitro study that investigated the possible anti-inflammatory and chondroprotective effects of celecoxib plus GS in human OA chondrocytes [26]. In this study, the human OA chondrocytes were incubated with prescription-grade crystalline GS (pCGS), or celecoxib, or both. The findings showed that the combination of GS plus celecoxib significantly reduced gene expression and supernatant release of COX-2, prostaglandin E2, IL-1β, IL-6, TNF-α, MMP-1, MMP-3, MMP-13, while it increased type II collagen (Col2a1), in comparison with basal conditions or IL-1β stimulated cells. The authors further demonstrated a synergistic effect of celecoxib plus GS on OA chondrocyte apoptosis and oxidative stress and postulated that the observed effects were likely mediated by modulation of the nuclear factor (NF)-κB signaling pathway. This study indirectly supports the use of combination therapy of pCGS and celecoxib for the treatment of patients with OA [26].

KOA is a disease with a high rate of treatment failure, often with sub-optimal control of symptoms (i.e. persistent pain and physical impairment), ultimately leading to poor quality of life. A multimodal approach may be more effective in improving patient wellbeing and treatment success rates as compared to a single pharmacological agent. International guidelines [16] and global consensus documents [35–41] support this strategy. Further, the ESCEO algorithm recommends a stepwise multimodal approach, combining oral NSAIDs with SYSADOAs in patients with persistent symptoms. The combination of non-pharmacological and pharmacological approaches is the best option, and among the pharmacological therapies, add-on treatment with drugs working synergistically can provide better control. The findings of this scoping review support the use of a multimodal approach, using the combination of background therapy with GS plus an NSAID to manage symptoms related to KOA and to delay disease progression, with a potential benefit on the joint structure. However, treatment decisions should be made by the physician according to each patient’s individual risk/benefit profile (e.g., celecoxib is suggested for up to 30 days in patients with increased CV risk, whereas nonselective NSAIDs should be given for less than 7 days). Further, treatment decisions should also take account of patients’ preferences [42, 43].

This review has some limitations. First, we searched only the published literature in PubMed, Cochrane and Embase, and we did not search unstructured databases or the grey literature. As a result, only 5 studies, with limited sample sizes were identified and included in the review. Additionally, among the eligibility criteria, we selected only studies published in the English language. We cannot rule out the possibility that informative studies published in languages other than English may have been overlooked. Second, the follow-up was short in all studies. Third, the treatment combinations varied in the studies included, and different molecules may have different effects on KOA symptomatology. Furthermore, only crystalline GS available as pCGS has been shown to have evidence-based clinical efficacy [44–46]. Finally, only two of the studies included in this review reported information about adverse events.

## Conclusions

This is the first scoping review to investigate existing literature about the potential benefits or synergistic effect of a combination of GS plus NSAIDs in KOA. This review provides an overview of 5 available clinical studies, which all reported a significantly greater benefit with combination therapy as compared to single-agent therapy, in terms of pain reduction, improvement in function and stiffness, and improvement in biomarkers of inflammation and cartilage degradation. Available data therefore suggest that the combination of GS and NSAIDs may be useful in a multimodal approach in patients with KOA. However, further randomized, controlled studies with larger sample sizes and longer-term follow-up are necessary to draw more robust conclusions about the efficacy and safety of this combination therapy, and to identify the best NSAID to associate with GS in this combination.

## Supplementary Information

**Additional file 1.**

## Data Availability

All data generated or analyzed during this study are included in this published article [and its supplementary information files].

## Abbreviations

Col2a1: Type II Collagen
COX-2: Cyclooxygenase 2 Inhibitors
CS: Chondroitin Sulfate
CTX-I: C-terminal telopeptide type 1
CTX-II: C-terminal telopeptide type 2
CV: Cardiovascular
ESCEO: European Society for Clinical and Economic Aspects of Osteoporosis, Osteoarthritis and Musculoskeletal Diseases
GI: Gastrointestinal
GS: Glucosamine Sulfate
IL: Interleukin
JNK: C-Jun N-terminal kinase
KOA: Knee Osteoarthritis
LKSS: Lysholm Knee Score Scale
MMP: Matrix Metalloproteinase
NF: Nuclear Factor
NO: Nitric Oxide
NSAIDs: Non-Steroidal Anti-Inflammatory Drugs
OA: Osteoarthritis
pCGS: Prescription-Grade Crystalline GS
PGE2: Prostaglandin-2
PRISMA: Preferred Reporting Items for Systematic reviews and Meta-Analyses
RCT: Randomized Control Trials
SOD: Superoxide Dismutase
SYSADOAs: Symptomatic Slow-Acting Drugs for Osteoarthritis
TNF-α: Tumor Necrosis Factor α
VAS: Visual Analogue Scale
WOMAC: Western Ontario and McMaster Universities Arthritis Index
