# Supplementary material for: Possible synergic action of non-steroidal anti-inflammatory drugs and glucosamine sulfate for the treatment of knee osteoarthritis: a scoping review
Source: BMC Musculoskelet Disord. 2022 Dec 12;23:1084. doi: 10.1186/s12891-022-06046-6 (PMC9743630; doi:10.1186/s12891-022-06046-6)
Supplement: Supplementary file 1 — Additional file 1. [file 12891_2022_6046_MOESM1_ESM.docx]

**SYNERGIC ACTION OF NON-STEROIDAL ANTI-INFLAMMATORY DRUGS AND GLUCOSAMINE SULFATE FOR THE TREATMENT OF KNEE OSTEOARTHRITIS: A SCOPING REVIEW**

Nicola Veronese^1^, Fiona Ecarnot^2*^, Sara Cheleschi^3^, Antonella Fioravanti^3^,Stefania Maggi^4^

**Affiliation**:

^1^Department of Internal Medicine, Geriatrics Section, University of Palermo, Palermo, Italy.

^2^University Hospital, and Research unit EA3920, University of Franche-Comté, 25000 Besançon, France

^3^Rheumatology Unit, Department of Medicine, Surgery and Neuroscience, Azienda Ospedaliera Universitaria Senese, Policlinico Le Scotte, 53100 Siena, Italy

^4^National Research Council, Neuroscience Institute, Aging Branch, Padova, Italy.

*Corresponding author: Fiona Ecarnot, PhD

**Supplementary Appendix**

**Supplementary Table 1** Search strategy for Medline/ PubMed

| **Description** | **#** | **Search string** | **Results** |
| --- | --- | --- | --- |
| Glucosamine sulfate | 1 | (“2-Amino-2-Deoxyglucose” [tiab] OR “2 Amino 2 Deoxyglucose” [tiab] OR “Glucosamine Sulfate” [tiab] OR Glucosamine [mh]) | 15,130 |
| NSAIDS | 2 | (“Non steroidal Anti-inflammatory drugs*” [tiab] OR NSAID* [tiab] OR “Non steroidal Anti-inflammatory agents*” [tiab] OR “Non steroidal Anti inflammatory drugs*" [tiab] OR “Non steroidal Anti inflammatory agents*” [tiab] OR “Anti Inflammatory Agents, Non-Steroidal" [Mesh] OR Celecoxib* [tiab] OR “SC 58635” [tiab] OR “SC-58635” [tiab] OR “SC58635” [tiab] OR "Celecoxib"[Mesh] | 104,493 |
| Human beings | 3 | “Human beings*” [tiab] OR Humans* [tiab] OR “Homo sapiens*” [tiab] OR "Humans"[Mesh] | 20,546,527 |
| Total combined results | 4 | #1 AND #2 AND #3 AND #4 | 221 |

**Supplementary Table 2** Search strategy for Embase

| **Search string** | **Results** |
| --- | --- |
| 1.cyclooxygenase 2 inhibitor/  2. Cyclooxygenase 2 Inhibitors.mp 3. COX-2 Inhibitor.mp.  4. COX 2 Inhibitor.mp.  5. COX-2 Inhibitors.mp.  6. COX 2 Inhibitors.mp.  7. coxib.mp. 8. Etodolac.mp. or etodolac/  9. celecoxib/ or Celecoxib.mp.  10. Etodolac.mp. or etodolac/  11. Etoricoxib.mp. or etoricoxib/  12. ibuprofen/ or Ibuprofen.mp.  13. Lumiracoxib.mp. or lumiracoxib/  14. meloxicam/ or Meloxicam.mp.  15. Nabumetone.mp. or nabumetone/  16. Parecoxib.mp. or parecoxib/  17. Polmacoxib.mp. or polmacoxib/  18. Rofecoxib.mp. or rofecoxib/  19. 1 or 2 or 3 or 4 or 5 or 6 or 7 or 8 or 9 or 10 or 11 or 12 or 13 or 14 or 15 or 16 or 17 or 18  20. 2-Amino-2-Deoxyglucose.mp.  21. 2 Amino 2 Deoxyglucose.mp.  22. Glucosamine Sulfate.mp.  23. exp glucosamine sulfate/  24. 20 or 21 or 22 or 23  25. 19 and 24 | 21196  1430  6061  6061  6023  6023  564  2853  23788  2853  3154  54696  1184  8320  2085  2084  16  10649  100876  17  17  1271  1149  1288  309 |
| Number of search hits | 309 |

**Supplementary Table 3** Search strategy for Medline/ Cochrane

| **#** | **Search string (**in Title Abstract Keyword) | **Results** |
| --- | --- | --- |
| 1 | Glucosamine sulfate AND celecoxib | 43 |
| 2 | Glucosamine sulfate AND Nonsteroidal inflammatory drugs | 27 |
| 3 | Glucosamine sulfate AND Nonsteroidal anti-inflammatory agents | 16 |
| 4 | Glucosamine sulfate AND NSAIDs | 43 |
| 5 | Total search hits | **129** |
